# A Low-Cost Simulation Model for R-Wave Synchronized Atrial Pacing in Pediatric Patients with Postoperative Junctional Ectopic Tachycardia

**DOI:** 10.1371/journal.pone.0150704

**Published:** 2016-03-04

**Authors:** Andreas Entenmann, Ronald Schmiedel, Miriam Michel, Friedemann Egender, Vera Heßling, Ingo Dähnert, Roman Gebauer

**Affiliations:** 1 Department of Pediatrics, Innsbruck Medical University, Innsbruck, Austria; 2 Department of Pediatric Cardiology, University of Leipzig, Leipzig, Germany; 3 Department for Congenital Heart Disease and Pediatric Cardiology, Schleswig-Holstein University Hospital, Kiel, Germany; University of Tampere, FINLAND

## Abstract

**Background:**

Postoperative junctional ectopic tachycardia (JET) occurs frequently after pediatric cardiac surgery. R-wave synchronized atrial (AVT) pacing is used to re-establish atrioventricular synchrony. AVT pacing is complex, with technical pitfalls. We sought to establish and to test a low-cost simulation model suitable for training and analysis in AVT pacing.

**Methods:**

A simulation model was developed based on a JET simulator, a simulation doll, a cardiac monitor, and a pacemaker. A computer program simulated electrocardiograms. Ten experienced pediatric cardiologists tested the model. Their performance was analyzed using a testing protocol with 10 working steps.

**Results:**

Four testers found the simulation model realistic; 6 found it very realistic. Nine claimed that the trial had improved their skills. All testers considered the model useful in teaching AVT pacing. The simulation test identified 5 working steps in which major mistakes in performance test may impede safe and effective AVT pacing and thus permitted specific training. The components of the model (exclusive monitor and pacemaker) cost less than $50. Assembly and training-session expenses were trivial.

**Conclusions:**

A realistic, low-cost simulation model of AVT pacing is described. The model is suitable for teaching and analyzing AVT pacing technique.

## Introduction

Postoperative junctional ectopic tachycardia (JET) is a serious complication after pediatric cardiac surgery [1]. It is a narrow complex tachycardia due to an autonomous focus at the Bundle of His. Typically the ventricular heart rate is higher or equal to the atrial rate. The tachycardia and the loss of atrioventricular synchrony may severely reduce cardiac output [2]. Therefore the arrhythmia is associated with increased morbidity and mortality [3, 4].

**Fig 1:**
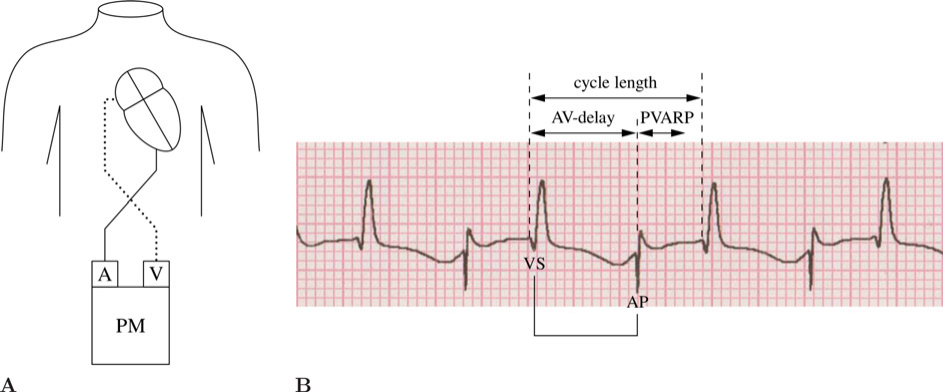
Principles of AVT pacing. (A) External pacing wires are switched at the pacemaker´s inputs. The pacemaker senses ventricular depolarization via the atrial channel and stimulates the atria before the next QRS complex, modified from [6]. (B) Original electrocardiogram during AVT pacing in a 3-month-old child with postoperative JET. A: atrial input, V: ventricular input, PM: external pacemaker, VS: ventricular sensing, AP: atrial pacing, AV: atrioventricular, PVARP: postventricular atrial refractory period.

Therapy for postoperative JET comprises the administration of anti-arrhythmic drugs, weaning of catecholamines, deep sedation, and induced hypothermia [5]. Different techniques of cardiac pacing are used aiming either to restore atrioventricular synchrony or to reduce the junctional heart rate [6]. R-wave synchronized atrial pacing is an innovative temporary pacing technique used to reconstitute atrioventricular synchrony without altering patient heart rate. The method was first described by Till and Roland in 1991 and made generally applicable by Janoušek et al. in 2003 [7, 8]. The basic idea of this method is stimulation of the atria when triggered by a sensed ventricular action (Fig 1). According to Generic Pacemaker Code, R-wave synchronized atrial pacing can be described as AVT pacing, as the atria are stimulated, the ventricles are sensed, and the mode is *triggered* [9].

Its benefits made AVT pacing the first-line strategy in patients with postoperative JET at our department. Between June, 2009, and December, 2011, however, our pediatric cardiologists abandoned AVT pacing as the principal pacing strategy in 7 of 26 instances of JET (27%). We therefore sought to design and to evaluate a simulation model for R-wave synchronized atrial pacing in order to identify technical problems leading to user drop-out and to establish a tool for standardized training.

## Methods

### JET simulator

**Fig 2:**
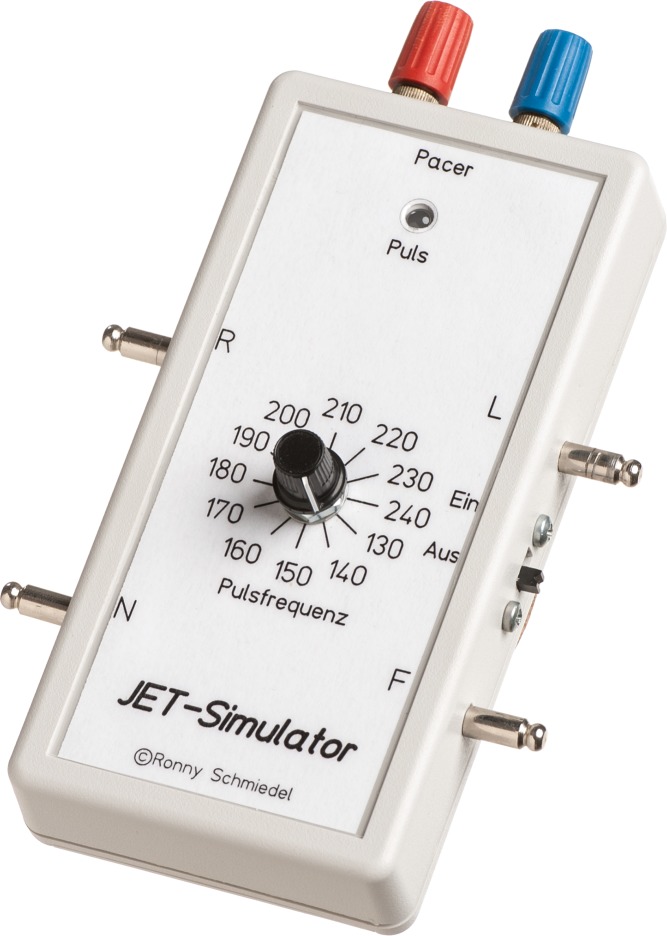
The custom-made JET simulator. JET rates are selected via a rotary switch in the middle of one face. Signals for an ECG monitor are provided at the lateral output sites. Output plugs at the top supply the input signal for the pacemaker. An on/off switch is located at the right side of the device. R: right, L: left, F: foot, N: neutral.

An 8-bit microcontroller (Atmel, San Jose, CA) forms the core of this system. The electrocardiogram (ECG), without P-wave, is digitized and stored as an array in flash, where it is read using various sampling rates, depending on the pulse rate desired. The ECG curve is created using pulse width modulation and a downstream 2-in-1 low pass filter with a cut-off-frequency of 159 Hz. Subsequent voltage dividers enable splitting and weighting the outputs in various different ways. This provides the ECG signal in the form of right arm (R), left arm (L), right foot (F), and neutral (N) signals to the outputs on the side of the simulator. Furthermore, impedance is adjusted to ensure suitability as pacemaker input signal (on the top of the simulator, Fig 2).

The various JET rates are selected in increments of 10 bpm within a frequency range of 130 to 240 bpm. The LED above the rotary switch flashes in time with the simulated heart rate. The device is powered by two microcells and an integrated step-up converter, to achieve the required operating voltage of 3.3 V. A reverse polarity protection prevents damage should the batteries be inserted the wrong way round.

The costs of the materials required are less than $50. The circuit diagram and component list are available on request.

### Simulation model

**Fig 3:**
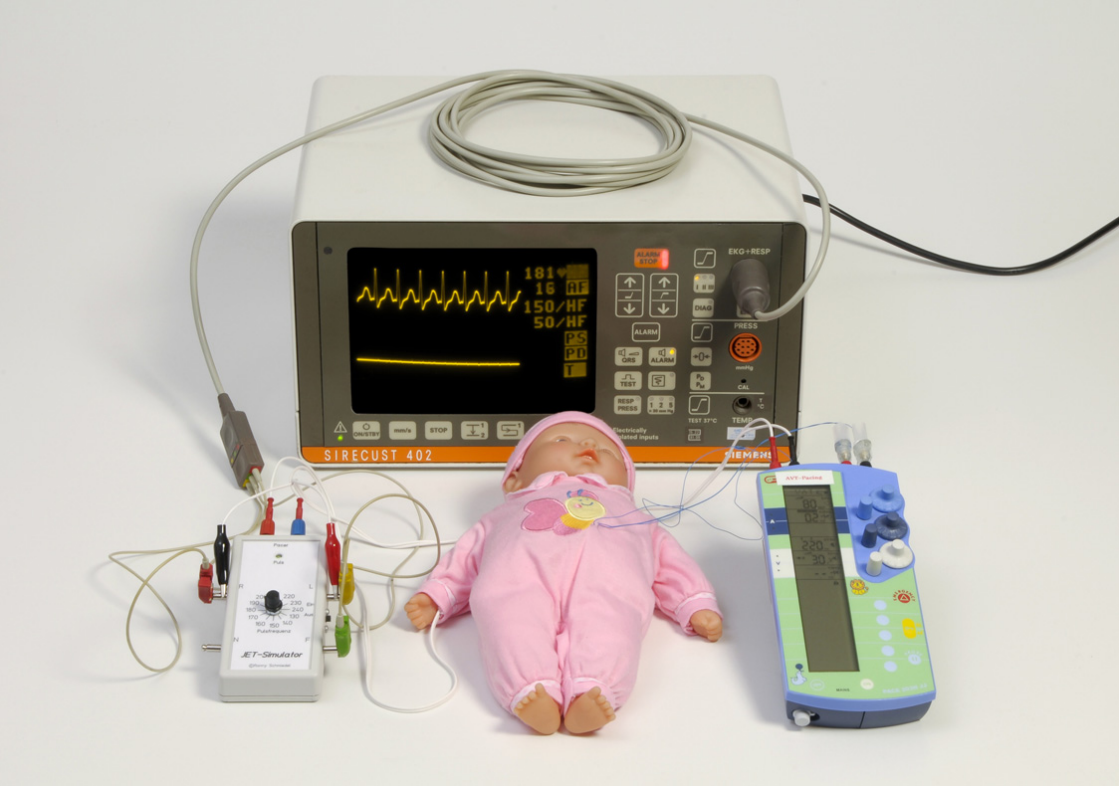
The simulation model. The model is composed of the JET-Simulator, an ECG monitor, a simulation doll, and an external AVT capable pacemaker. The doll is fitted with atrial and ventricular external pacing wires, simulating an infant after cardiac surgery.

The custom-made JET simulator is connected to a commercial ECG monitor (Sirecust 402, Siemens, Munich, Germany) via the three output sites R, L, and F (with red, yellow, and green clips). Thus the monitor shows a typical ECG tracing of postoperative JET with narrow QRS complexes and no visible P-waves with the pre-selected heart rate in real time (Fig 3).

The signals for the pacemaker are transferred to a doll using two connecting wires. Inside the doll these wires are linked with temporary ventricular pacing wires (unipolar temporary myocardial electrodes, TME Z; Osypka Medical, La Jolla, CA). The connectors of the ventricular pacing wires are then plugged into the atrial inputs of an external pacemaker suitable for AVT pacing (Osypka PACE 203 H). Atrial temporary pacing wires are connected to the ventricular inputs of the external pacemaker and linked via a 2000 Ohm resistance circuit inside the doll´s body in order to avoid activating pacemaker disconnection alarms.

### Test record

Ten fully trained pediatric cardiologists well-experienced in AVT pacing were invited to test using of the simulator under laboratory conditions. The testers were presented with the testing environment described above; however, the four temporary pacing wires were not yet connected to the pacemaker and the pacemaker was not yet switched on. The JET simulator was set to a heart rate of 200 bpm. The cardiologists were then asked to establish AVT pacing. Each single step necessary to perform effective AVT pacing was included in a test protocol. This protocol contained a total of 10 different working steps: (ON) switching on the device, (VDD) choosing the dual sensing and ventricular pacing mode, (V-SENSE) selecting maximal insensitivity of ventricular sensing, (MTR) setting the maximum tracing rate 10–20 bpm higher than the JET rate, (AV-DLY) adjusting the atrioventricular delay to the highest possible value, (PVARP) selecting a postventricular atrial refractory period of 100 ms, (RATE) setting the basic stimulation rate at a value clearly below the JET rate, (R-WAVE) measuring the ventricular input signal, (A-SENSE) tuning atrial sensing to 50% of R-WAVE, and (WIRES) connecting the temporary pacing wires. We used these default settings: MTR 230 bpm, RATE 210 bpm, PVARP 200 ms, AV-DLY 50 ms, ventricular stimulation voltage (V-STIM) 12 V, and V-SENSE and A-SENSE each 8 mV.

**Fig 4:**
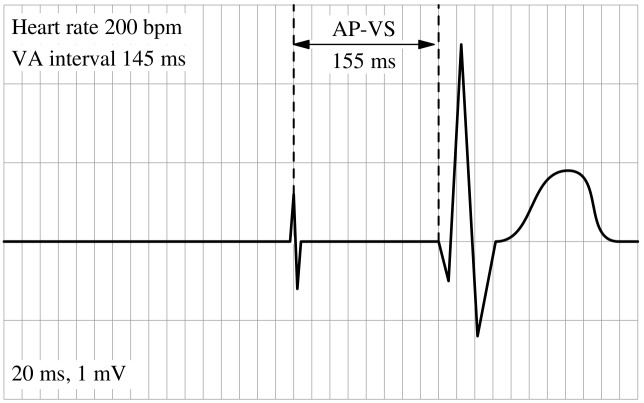
The MetaPost AP-VS-Visualizer. A short software script utilizes the patient´s heart rate (JET simulator rate) and the AV delay as adjusted by the tester to calculate and graphically to display the interval between atrial pacing and sensing of the subsequent QRS complex. AP: atrial pacing, VS: ventricular sensing, AV: atrioventricular, bpm: beats per minute, ms: milliseconds, mV: millivolt.

For each of the 10 working steps the investigators scored tester performance as earning 1, 2, or 3 points (major mistake, minor mistake, correct). A major mistake was defined as a failure that impeded effective or safe AVT pacing. A minor mistake was defined as an approach deviating from protocol while not necessarily impeding effective or safe AVT pacing. Each test session was video recorded. To illustrate the results of the adjustments made by the testers we used a custom-made software script based on the free programming language and interpreter MetaPost by J.D. Hobby (Fig 4).

Each cardiologist was asked in a debriefing session after the test whether they considered the testing environment realistic, whether they had improved their own skills during the test, and whether they think that the simulation model is a valuable tool for training pediatric cardiologists or pediatric intensive-care physicians in AVT pacing. The cardiologists framed their answers using a five-point Likert scale (1 = absolutely not, 5 = absolutely yes).

### Ethics

The development of the simulation model, testing, and training was part of a quality assurance program within the scope of the AVTPS study. This study was approved by the institutional review board (ethics commission) of the Medical Faculty of Christian-Albrechts-University Kiel (A 113/2). Specific information about the simulation test was not provided. All volunteers taking part in the simulation test were informed verbally before the test started that data were intended for publication in a peer-reviewed scientific journal. In a debriefing session after the test, all testers were asked to evaluate the simulation model. An evaluation form was handed out. This form contained written information that taking part in the study was voluntary and that data were encoded and stored anonymously (S1 File). Receipt of the filled-in evaluation forms was taken as receipt of written informed consent. All personal data, including the video clips, were removed after analysis was completed.

## Results

### Evaluation

Ten fully trained pediatric cardiologists tested the simulator. Their experience in pediatric cardiology ranged from 4 to 14 years (median 5). All testers evaluated the testing environment as realistic (6/10) or very realistic (4/10). Nine declared that they had improved their skills during the test. All considered the simulator as a valuable or very valuable tool for training AVT pacing.

### Performance Analysis

**Fig 5:**
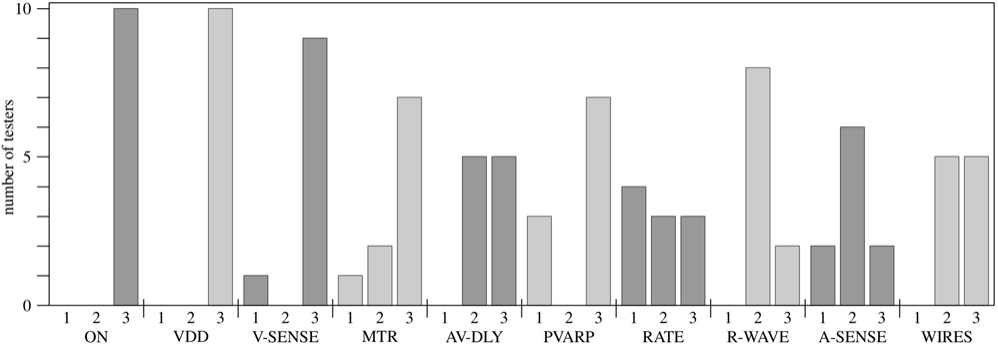
Simulator testing. Ten pediatric cardiologists were asked to establish AVT pacing. Ten working steps were assessed: ON, switching on the pacemaker; VDD, choosing the VDD mode; V-SENSE, adjusting ventricular sensing for maximal insensitivity; MTR, selecting the maximal tracking rate at a value 10–20 bpm above the patient´s heart rate; AV-DLY, setting the AV delay to the maximum allowed value; PVARP, adjusting the post ventricular atrial refractory period to 100 ms; RATE, selecting a basic stimulation rate clearly below the patient´s heart rate; R-WAVE, measuring the ventricular input signal; A-SENSE, selecting an atrial sensitivity 50% of the ventricular input signal; WIRES, connecting the pacing wires of the pacemaker. Three points indicate perfect, 2 points suboptimal performance, and 1 point a mistake that impairs safe or effective AVT pacing.

None of the 10 testers had problems switching on the pacemaker device (ON) or choosing the VDD mode (VDD). One tester neglected to set ventricular sensing for maximal insensitivity (V-SENSE). Thus the pacemaker did not switch into VAT mode. Seven testers adjusted the MTR correctly to a value of 10–20 bpm above the monitor rate (MTR, 3 points). Two adjusted the MTR to a rate equal to the monitor rate, thus allowing very short AP-VS intervals that may not be hemodynamically beneficial (2 points). One tester did not at all alter the MTR from its default value of 230 bpm. The maximum possible AV delay was consequently restricted to 60 ms. This setting may not be hemodynamically favorable (1 point). Five of the testers adjusted the AP-VS interval indirectly by selecting the pacemaker’s maximum possible AV delay (AV-DLY, 3 points). The other half used individual adjustments (2 points). Three testers neglected to set the PVARP of the pacemaker to 100 ms, thus impeding effective AVT pacing (PVARP, 1 point). Only 3 testers set the basic stimulation rate of the pacemaker to values clearly below the monitor heart rate (RATE, 3 points). Another 3 testers used adjustments that may cause inconvenient atrial stimulations if the patient´s heart rate decreases more than 20 bpm in the further course of the arrhythmia (2 points). Four testers did not change the basic stimulation rate from its default setting of 210 bpm. The pacemaker will stimulate the atria, without ventriculoatrial synchronization, at 210 bpm in this setting (1 point). Eight testers did not measure the voltage of the ventricular input signal via the atrial channel of the pacemaker using the pause function (R-WAVE). This deviates from protocol (2 points) but does not necessarily cause problems if the sensitivity for the ventricular input signal is set correctly in the next step. Three testers did indeed adjust the atrial sensitivity (effective ventricular sensitivity) correctly to 50% of the measured signal strength (A-SENSE). Six used other adjustments that allowed effective sensing of the ventricular signal (2 points). Two testers did not alter the default setting (8 mV), thus impairing ventricular sensing via the atrial channel (1 point). All testers connected the pacing wires in the correct manner, in which the ventricular pacing wires are linked with the atrial plugs of the pacemaker and the atrial pacing wires with the ventricular plugs (WIRES). Only half of the subjects connected the ventricle wires first, as safety requires (3 points). The results of the simulation test are summarized in Fig 5.

## Discussion

In the management of postoperative junctional ectopic tachycardia, different temporary pacing techniques aim either to restore atrioventricular synchrony or to reduce the patient´s heart rate [6, 10]. In this context atrial demand pacing (AAI) is most commonly used to overdrive the patient´s ventricular heart rate and to re-establish atrioventricular synchrony. The disadvantage of AAI pacing is that a stimulation rate even higher than the patient´s heart rate is required [6]. AAI pacing further relies on intact atrioventricular conduction; this is often disturbed in patients with JET [11]. R-wave synchronized atrial pacing is an innovative and valuable way to restore atrioventricular synchrony in pediatric patients with postoperative JET. The main advantages of AVT pacing are that it does not alter the patient´s heart rate and that it is independent of atrioventricular conduction. Janoušek et al. showed positive effects on blood pressure in 9 patients treated with AVT pacing [8]. The authors concluded their study with the statement that AVT pacing should be incorporated into the standard treatment protocol for postoperative JET. Our study shows, however, that AVT pacing is complex, with several technical pitfalls that may impede effective synchronized pacing. The user has to carry out a collection of tasks, divided into 10 working steps in our simulation model, if AVT is to be successful. Our analysis identified 5 working steps (V-SENSE, MTR, PVARP, RATE, A-SENSE) in which at least one of 10 testers made errors that impaired effective and safe AV synchronized pacing. Some of the mistakes may be oversights, like neglecting to select maximal insensitivity for ventricular sensing in the V-SENSE step. Others demonstrate that the technical details of this form of pacing are difficult to understand. The switch of the pacing wires entails substantial changes in pacemaker function and nomenclature. The postventricular atrial refractory period (PVARP), for example, becomes the postatrial ventricular refractory period, ventricular sensing becomes atrial sensing, and the maximum tracking rate serves as a reference rate in defining input requirements for the maximum length of the adjustable AV delay. We identified understanding of the basic stimulation rate in the RATE step as specifically problematic. This stimulation rate has no particular function in AVT pacing but may disturb atrial pacing if it is higher than the patient´s heart rate. We recommend adjustment of the basic stimulation rate to 100 bpm (clearly below the JET rate). Our findings led us to introduce at our institution a defined, specific standard operating procedure for AVT pacing with detailed instructions on necessary settings.

Our study overall permitted us to demonstrate that a realistic simulation model for R-wave synchronized atrial pacing can be established at low cost. All testers assessed the simulation environment as realistic. All but one claimed that they gained knowledge and understanding of the technique and improved their skills by taking part in the test, and all assessed the simulation model as a valuable tool in teaching pediatric cardiologists or pediatric intensive-care physicians the technique of AVT pacing.

The simulator can be improved, for example by representation of the atrial pacing spike on the monitor. We plan to refer the atrial stimulus from the pacemaker to the ECG monitor after reducing voltage and current. Effects of changes in AV delay thus can be assessed at the monitor in real time. Further possibilities include placement of the JET simulator within the simulation doll and remotely controlled, continuous adjustment of the JET heart rate, as well as incorporation of ECG plugs into the doll’s body to simulate the clinical setting more realistically.

## Conclusion

We established a new, low-cost simulation of R-wave synchronized atrial pacing in an infant with postoperative JET. The model proved suitable for teaching the technique and for analyzing user performance. Several technical pitfalls were identified that may impede effective and safe AVT pacing; setting of the basic stimulation rate in particular was found to be a major source of error. Evaluators of the simulation assessed it as realistic, valuable, and usable.

## Supporting Information

S1 File Evaluation Form. (PDF)
