# Supplementary material for: A Low-Cost Simulation Model for R-Wave Synchronized Atrial Pacing in Pediatric Patients with Postoperative Junctional Ectopic Tachycardia
Source: PLoS One. 2016 Mar 4;11(3):e0150704. doi: 10.1371/journal.pone.0150704 (PMC4778927; doi:10.1371/journal.pone.0150704)
Supplement: S1 File — (PDF) [file pone.0150704.s001.pdf]

Evaluationsbogen

Evaluation form

Liebe Kollegin, lieber Kollege!

Vielen Dank für die Teilnahme an unserem AVT-Pacing Simulationstest. Wir würden gerne einen Artikel über das Simulationsmodell in einer wissenschaftlichen und begutachteten Zeitschrift veröffentlichen. Dazu wollen wir auch die Daten aus dem Simulationstest verwenden. Zusätzlich benötigen wir Einschätzung der Testpersonen zur Beurteilung der Qualität unseres Modells. Wir bitten Euch daher uns bei diesem Projekt zu unterstützen, den Evaluationsbogen auszufüllen und in mein Fach im Erdgeschoss (Andreas Entenmann) zu werfen. Die Teilnahme an der Studie und an der Evaluation ist freiwillig und anonym. Nach der Auswertung werden sämtliche personenbezogene Daten und die Videos vom Server gelöscht. Für Eure Hilfe danke ich Euch herzlich!

Andreas Entenmann

Dear collegues,

Thank you very much for taking part in our AVT-pacing simulation test. We plan to publish an article about the simulation model in a peer-reviewed scientific journal. Therefore we intend to use the data of the simulation test. Furthermore we need the evaluation of the testers concerning the quality of our model. Thus, we would like to ask for your support of our project. Please fill out this evaluation form and return it to my post box on the ground floor (Andreas Entenmann). Taking part in the study and it´s evaluation is voluntarily and anonymous. All personal data will be deleted from the server after analysis, including the video sequences. Thank you very much for your kind help!

Andreas Entenmann

Im Folgenden werden drei Aussagen gegeben. Bitte bewertet diese Aussagen auf einer fünfstufigen Skala. Die Bedeutung der Stufen ist dabei wie folgt:

1 = die Aussage trifft überhaupt nicht zu
2 = die Aussage trifft eher nicht zu
3 = die Aussage ist weder richtig noch falsch
4 = die Aussage trifft eher zu
5 = die Aussage trifft absolut zu

Three statements are given below. Please evaluate these statements using a 5-step evaluation scale. The meaning of each step is as follows:

1 = the statement is absolutely wrong
2 = the statement is rather inaccurate
3 = the statement is ambivalent
4 = the statement is rather accurate
5 = the statement is absolutely correct

1. Aussage:
„Das Modell bietet die realistische Simulation eines Säuglings mit postoperativer junctional ectopen Tachycardie (Umgebung und Therapie).“

1st statement:
„The model provides a realistic simulation scenario of an infant suffering from postoperative junctional ectopic tachycardia (setting and therapy).“

☐	☐	☐	☐	☐
1	2	3	4	5

2. Aussage:
„Mit der Teilnahme am Simulationstest habe ich mein Wissen und meine Fähigkeiten bei der Durchführung des AVT-Pacings verbessern können.“

2nd statement
„Taking part in the simulation test I personally improved my knowledge and skills in performing AVT-pacing.“

☐	☐	☐	☐	☐
1	2	3	4	5

3. Aussage:
„Ich halte das Simulations-Modell für geeignet und wertvoll für das Training von Kinderkardiologen und Kinderintensivmedizinern beim Erlernen des AVT-Pacings.“

3rd statement:
„This simulation model is a suitable and valuable training tool for pediatric cardiologists and pediatric intensive care physicians intending to learn the technique of AVT-Pacing.“

☐	☐	☐	☐	☐
1	2	3	4	5
